# Incidence of Y-chromosome microdeletions in children whose fathers underwent vasectomy reversal or *in vitro* fertilization with epididymal sperm aspiration: a case-control study

**DOI:** 10.1590/S1679-45082016AO3805

**Published:** 2016

**Authors:** Milton Ghirelli-Filho, Patricia Leme de Marchi, Fernanda Abani Mafra, Viviane Cavalcanti, Denise Maria Christofolini, Caio Parente Barbosa, Bianca Bianco, Sidney Glina

**Affiliations:** 1 Faculdade de Medicina do ABC, Santo André, SP, Brazil.; 2 Hospital Israelita Albert Einstein, São Paulo, SP, Brazil.

**Keywords:** Chromossomes, human, Y, Vasectomy, Fertilization *in vitro*, Reproductive techniques

## Abstract

**Objective:**

To evaluate the incidence of Y-chromosome microdeletions in individuals born from vasectomized fathers who underwent vasectomy reversal or *in vitro* fertilization with sperm retrieval by epididymal aspiration (percutaneous epididymal sperm aspiration).

**Methods:**

A case-control study comprising male children of couples in which the man had been previously vasectomized and chose vasectomy reversal (n=31) or *in vitro* fertilization with sperm retrieval by percutaneous epididymal sperm aspiration (n=30) to conceive new children, and a Control Group of male children of fertile men who had programmed vasectomies (n=60). Y-chromosome microdeletions research was performed by polymerase chain reaction on fathers and children, evaluating 20 regions of the chromosome.

**Results:**

The results showed no Y-chromosome microdeletions in any of the studied subjects. The incidence of Y-chromosome microdeletions in individuals born from vasectomized fathers who underwent vasectomy reversal or *in vitro* fertilization with spermatozoa recovered by percutaneous epididymal sperm aspiration did not differ between the groups, and there was no difference between control subjects born from natural pregnancies or population incidence in fertile men.

**Conclusion:**

We found no association considering microdeletions in the azoospermia factor region of the Y chromosome and assisted reproduction. We also found no correlation between these Y-chromosome microdeletions and vasectomies, which suggests that the assisted reproduction techniques do not increase the incidence of Y-chromosome microdeletions.

## RESUMO

**Objetivo:**

Avaliar a incidência de microdeleções do cromossomo Y em indivíduos nascidos de pais vasectomizados submetidos à reversão de vasectomia ou fertilização *in vitro* com recuperação de espermatozoides por aspiração do epidídimo (aspiração percutânea de espermatozoides do epidídimo).

**Métodos:**

Estudo caso-controle que compreende crianças do sexo masculino de casais em que o homem havia sido previamente vasectomizado e escolheu reversão da vasectomia (n=31) ou fertilização *in vitro* com recuperação espermática por aspiração percutânea de espermatozoides do epidídimo (n=30) para obtenção de novos filhos, e um Grupo Controle de crianças do sexo masculino de homens férteis com vasectomia programada (n=60). A pesquisa de microdeleções do cromossomo Y foi realizada por reação em cadeia da polimerase nos pais e filhos, avaliando 20 regiões do cromossomo.

**Resultados:**

O resultado não revelou microdeleções do cromossomo Y em qualquer indivíduo estudado. A incidência de microdeleções do cromossomo Y em indivíduos nascidos de pais vasectomizados que sofreram reversão de vasectomia ou fertilização *in vitro* com espermatozoides recuperados pela aspiração percutânea de espermatozoides do epidídimo não diferiu entre os grupos, e não houve nenhuma diferença entre indivíduos controle nascidos de gestações naturais ou incidência populacional em homens férteis.

**Conclusão:**

Não foi encontrada nenhuma associação considerando microdeleções da região do fator de azoospermia no cromossomo Y e reprodução assistida. Não houve correlação entre microdeleções do cromossomo Y e vasectomia, o que sugere que as técnicas de reprodução assistida não aumentam a incidência de microdeleções do cromossomo Y.

## INTRODUCTION

Assisted reproductive techniques (ART) are currently available worldwide and are successfully practiced on a large scale. Approximately 1 to 4% of all births in Europe are the result of *in vitro* fertilization (IVF), by both classical and intracytoplasmic sperm injection technique (ICSI).^(1)^

Among the factors that may cause male infertility and lead to indication of IVF are genetic anomalies, such as chromosomal aberrations, which are found in 5 to 15% of infertile men (5 to 7% in oligozoospermic and 10 to 15% in azoospermic men), and Y-chromosome microdeletions (YCM), which is one of the causes of severe testiculopathy, present in 2% of infertile men.^(2)^ Another important factor that leads to male infertility and indicates IVF is the obstructive azoospermia secondary to vasectomy, which represents a particular problem regarding possible defects associated with IVF implementation, since the consequences of obstruction on spermatogenesis are not yet completely known. Vasectomy is the most common cause of obstructive azoospermia and its prevalence varies from 7 to 10% of couples, and it increases with age: approximately 18% of men resort to vasectomies by the age of 45.^(3)^

Fertility recovery after a vasectomy is desired by 2 to 10% of men, and two methods are used for this purpose: vasectomy reversal or IVF with percutaneous epididymal sperm aspiration (PESA).^(3,4)^Vasectomy reversal has established itself as a very effective method for obtaining fertility after the procedure, and it was the only treatment option for vasectomized patients until the development of IVF using ICSI.^(4)^

Since then, both methods are available for any vasectomized individual, and the success rates of each method depend on many variables. It is up to the couple and their physician to decide on the most appropriate method. However, the possibility of a greater number of malformations in the offspring of patients who undergo IVF is still under discussion, which may influence the decision about the method used to recover fertility after vasectomy.^(3,4)^

Although ART has given infertile males with azoospermia and oligozoospermia the opportunity to conceive a child, this group has a great chance of having offspring with congenital malformations and/or genetic disorders.^(5-8)^ Some researchers questioned the genetic implications of ART for the offspring,^(9-12)^ and suggested higher incidences of fetal sexual and chromosomal aberrations, chromosomal anomalies due to *de novo* mutations, gene mutation and aneuploidy in spermatozoa.

Considering the genetic factors, YCM are a common identifiable cause of spermatogenesis failure. The vast majority of these microdeletions appear in *de novo* mutation, indicating that this region is particularly unstable.^(13,14)^

The incidence of YCM in the infertile population is quite variable in different studies, ranging from 2.1 to 14% in azoospermic men, and 1.8 to 8.5 % in oligozoospermic men.^(2)^ Regarding the fertile population, the incidence of YCM is a subject of discussion, since the classification of ‘fertile’ does not imply normozoospermia, which can create biases. While in most studies the incidence of microdeletions in the normal population is 0%, Kent-First et al.,^(15)^ detected an incidence of eight individuals with YCM in a sample of 920 fertile men, which represents 0.87 % of cases that were taken into consideration.

In a study by Feng et al.,^(12)^ YCM was observed in 10.8 % of children originated from IVF. None of them were born from natural conception. The difference is statistically significant. The study sample comprised 37 cases in the group with people who were born from IVF, and 60 cases in the Control Group, in which children came from a natural conception.

In this context, the presence of a higher number of YCM in children conceived by vasectomized men, who underwent IVF with sperm retrieval by PESA, would increase the incidence of infertility in this population.

## OBJECTIVE

To evaluate the incidence of Y-chromosome microdeletions in individuals conceived by vasectomized fathers, who underwent vasectomy reversal or *in vitro* fertilization with percutaneous epididymal sperm aspiration.

## METHODS

The subjects included in the study were divided into 3 groups: Group A - sons of vasectomized fathers, who underwent vasectomy reversal, and whose mothers had natural pregnancies after the procedure; Group B - sons of vasectomized men, who underwent IVF with sperm retrieval by PESA; Group C (Control Group) - sons of fertile men who would undergo a vasectomy procedure.

In all groups the YCM research was performed with fathers and sons. The outcome variable assessed was the YCM incidence. The demographic variables analyzed were parental age at the pregnancy onset, the time between vasectomy and fertility recovery procedure, and the father age upon vasectomy.

### Patients

The patients included in this research were obtained from the *Centro de Estudos em Genética e Reprodução Humana da Faculdade de Medicina do* ABC, Santo André, Brazil.

This study included male children born from couples in which the man had been previously vasectomized and opted for vasectomy reversal, or IVF with sperm retrieval by PESA. We also selected children of fertile men who were programming vasectomy in order create a Control Group. Semen of the fertile men was collected before vasectomy for analysis.

Peripheral blood samples were collected after explaining the study objectives, providing information about the consent form; and/or after signing a term of agreement approved by the local ethics committee.

From the database of the organizations there were 815 candidates to enroll in the study so we could have potential participants for each of the three proposed groups.

### Group A (vasectomy reversal)

In Group A was included 278 patients who underwent surgery for vasectomy reversal. Among the individuals we were able to contact, 81 had at least one biological child. Among the children, 42 were male. Thirty one individuals went for data collection.

The mean age of 31 men at the time of the vasectomy reversal was 40.5±5.7 years, and the mean age of their wives upon completion of vasectomy reversal was 29.0±4.2 years.

The mean time elapsed between vasectomy and vasectomy reversal of the 31 individuals included in this study was 6.4±3.2 years. The mean time that the pregnancy occurred was 6.0±2.6 months after completion of vasectomy reversal. The mean age of men at the time of vasectomy was 34.1±5.8 years.

### Group B (IVF with PESA)

The study included 294 patients who underwent IVF with PESA. Among the individuals we were able to contact, 102 had at least one biological child. Among these children, 61 were male. Thirty individuals went to the data collection.

The mean age of the 30 men at the time of the IVF with PESA completion was 45.3±5.9 years, and the mean age of their wives upon completion of IVF with PESA was 32.9±3.5 years.

The mean time elapsed between the completion of vasectomy and IVF with PESA of the 30 subjects included in the study was 15.3±5.1 years. The mean age of the men at the time of vasectomy completion was 30.0±5.2 years.

### Comparison between Groups A and B

The data comparing Groups A and B are summarized in table 1.

**Table 1:** Comparison between Groups A and B: mean, standard deviation and p value

	Group A	Group B	p value*
Male age	40.5±5.72	45.3±5.87	0.002
Female age	29.0±4.17	32.9±3.51	<0.001
Time of vasectomy	6.42±3.21	15.3±5.07	<0.001
Age at vasectomy	34.1±5.81	30.0±5.18	0.005

* Statistical significance considered values below 0.05, Student *t* test.

### Group C (Control Group)

Blood samples were collected from 60 fertile patients who were scheduled to undergo vasectomy and authorized the blood sample collection in their male child.

The mean age of the 60 men was 38.7±6.2 years. In addition to the blood samples, sperm of all 60 men who were programming a vasectomy was collected before the procedure, for analysis. The data resulting from the sperm analysis are summarized in table 2. Sperm morphology was not included among the seminal parameters evaluated due to the wide variety of techniques used to evaluate sperm, hampering data comparability.

**Table 2:** Mean parameters of semen of fertile men included in the study

Volume (ml)	Concentration (millions per ml)	Motility		

		Progressive n (%)	Non-progressive n (%)	Immotile n (%)
2.83±1.31	72.1±44.5	65.6±15.6	7.94±3.30	27.8±10.03

Among the 60 fertile patients, 5.0% had some semen parameter out of the normalcy reference established by the World Health Organization (WHO), which means abnormal sperm concentration and motility. The mean age at vasectomy of Groups A and B was compared with the mean age of men in Group C. A statistically significant difference was observed in both Groups A and B when compared to the Control Group C (p<0.05).

### Y-chromosome microdeletion research

Blood sampling of the selected individuals was collected in an EDTA-containing tube to extract the DNA for the YCM research. Genomic DNA was extracted from peripheral blood lymphocytes according to the protocol of Lahiri et al.^(16)^

The Y chromosome *loci* involved in spermatogenesis (AZFa: sY746, SY84, sY86, DFFRY; AZFb: XKRY, sY118, sY113, sY127, sY134, sY143, RBM1Y and AZFc:, sY153, SY148, sY157, sY158, sY254, sY255, sY160 and the sex-determining gene SRY) were analyzed by polymerase chain reaction (PCR), according to the protocols modified by Umeno et al.^(17)^ and Mitra et al.^(18)^ The AMELX gene was used as internal control of successful amplification.

The PCR was performed with a final volume of 25μL containing 100ng of genomic DNA, 10mM MgCl2, 1.0mM dNTPs, 2.0U of Taq DNA polymerase (Invitrogen^®^), Tris-HCl (pH 8,4) 10mM, KCl 50mM, 2.0 mM of each primer. The reaction was processed in a thermocycler (Corbett Research, QIAGEN, Valencia, CA, USA) as follows: denaturation for 5 minutes, at 95°C, followed by 32 cycles of 50 seconds, at 95°C (denaturation), 45 seconds at 55°C or 59°C (annealing in accordance with standardization of each primer), and 45 seconds, at 72°C (extension) with a final extension of 5 minutes, at 72°C. The PCR products were submitted to agarose gel electrophoresis 2.5%, and stained with GelRed (Uniscience) (intercalating DNA).

Several precautions were taken to avoid false-positive results, including processing of all samples by a sole female operator, and the use of internal controls for the reaction (AMELX gene) as a positive control. Pre- and post-PCR workspaces were strictly separated, to avoid carryover of amplified DNA sequences to new PCR reactions.

### Semen analysis

Semen was collected from all patients in the Control Group in order to evaluate sperm quality, considering that even fertility being proven by biological children, we could not rule out seminal changes that could be associated with the YCM, which eventually would be found.

Semen samples were collected by masturbation, two to five days after the last ejaculation. The assessment of sperm concentration, sperm motility and ejaculate volume was performed as per the WHO^(19)^ criteria.

### Vasectomy reversal

The vasectomy reversal was performed by bilateral vasovasostomy in all cases; however, the surgical technique and the type of anesthesia varied according to surgeon’s preference.

### Epididymal aspiration

Epididymal sperm retrieval for IVF was performed by epididymis aspiration with hypodermic needle, with the patient under local anesthesia with lidocaine. The punctured area of the epididymis and the technique used also varied according to the surgeon’s preference.

### In vitro fertilization protocols

Ovarian stimulation of multiple follicles was carried out using purified or recombinant gonadotropins, and GnRH agonists or antagonists were used to suppress endogenous secretion of gonadotropins. Individual doses of each medication varied from case to case, taking into consideration the age of the wife and clinical exams. Patients who received hCG injection presented, at least, three follicles with about 17mm in diameter on ultrasonography, after ovarian stimulation. The oocyte aspiration guided by transvaginal ultrasonography was performed 36 hours after hCG injection.

Handling of the gametes was performed according to routine protocols, following the ICSI technique for IVF using semen retrieved by PESA.^(20-22)^

### Statistical analysis

Groups A and B were statistically compared regarding the variables age, time elapsed between vasectomy and fertility recovery procedure, and age upon vasectomy. These variables were analyzed by two-tailed Student’s *t* test. The level of significance was 5% with a statistical power of 80%.

The incidence of YCM observed in each group was compared, separately, to the average population estimated in several previous studies.^(14,23,24)^ Data on semen parameters and time between vasectomy reversal and pregnancy were only presented as mean and standard deviation, as they appeared in only one of the groups.

The calculated sample size for the variable incidence of YCM was 23 cases in each group. This evaluation was based on the data from previous subject studies.

## RESULTS

The samples of three groups were analyzed and no YCM were found in any individuals. The YCM incidence in individuals born from vasectomized fathers who underwent vasectomy reversal, or IVF with spermatozoa retrieved by PESA, did not differ between the groups, and it was not different from control individuals, born from natural pregnancies or from population incidence in fertile men. Data of the groups are summarized in table 3.

**Table 3:** Total number of subjects and number of subjects with Y-chromosome microdeletions in each group

	Number of individuals	Individuals with Y-chromosome
Group A		
Fathers	31	0
Children	31	0
Group B		
Fathers	30	0
Children	30	0
Group C		
Fathers	60	0
Children	60	0

## DISCUSSION

To knowledge, the present study is the first to compare YCM incidence in children of vasectomized patients, based on the techniques available to obtain fertility after vasectomy.

Previously, Lee et al.,^(25)^ screened for vertical transmission, expansion of microdeletions, and Y-chromosome *de novo* deletion in 33 male fetuses, conceived by 32 ICSI-treated patients. Y-chromosome was studied according to 10 Y-specific markers. The authors detected an overall microdeletion frequency of 12.5% in ICSI-treated patients (4 of 32 patients), in which all YCM were in the AZFc region.

Considering the fetuses, 11 out of 33 (33.3%) presented YCM. Two presented the same microdeletion as their fathers, two had larger microdeletions when compared to their fathers, which indicates expansion, and three presented a *de novo* YCM. These results support the view that Y-microdeletions can be transmitted and expanded through ICSI and appear as *de novo* in male offsprings.

Similarly, Feng et al.,^(12)^ investigated the chromosome mutation risks after ART for couples with comparable genetic backgrounds. Ninety-seven male children, whose fathers had normal spermatogenesis were studied, including 19 babies conceived through IVF, 18 through ICSI and 60 naturally conceived babies, as well as the babies’ fathers. The YCM were studied according to 13 Y-specific markers; and karyotype and neonatal examination were also considered in the study. The results showed that all children had a normal chromosomal composition, but *de novo* YCM in male children conceived through ICSI or IVF were statistically significantly higher than in those conceived naturally [10.8% *versus* 0, considering in 1 (5.3%) of 19 IVF offspring, and in 3 (16.7%) of 18 ICSI offspring], indicating that risks of gene mutation may increase in the ART offspring, although their fathers had normal spermatogenesis and genetic background.

Here, we evaluated the incidence of YCM in male children born from vasectomized fathers who underwent vasectomy reversal (n=31), or IVF with PESA (n=30), and from fertile men who were programming vasectomy (n=60). Y-chromosome was evaluated according to 20 Y-specific markers. The results showed no YCM in any studied subject. Y-chromosome microdeletions incidence in individuals born from vasectomized fathers who underwent vasectomy reversal, or IVF with spermatozoa retrieved by PESA, did not differ between the groups. Control subjects born from natural pregnancies or from population incidence in fertile men were not different, suggesting that the use of spermatozoa from PESA or vasectomy reversal did not increase the YCM incidence.

There is controversy regarding the fact of an increasing incidence of *de novo* YCM in offspring conceived via ART, especially ISCI. Nonetheless, the discrepancy observed in different studies may be the result of geographical and ethnical differences, inconsistent criteria in selection of patients, and several Y-chromosome markers screening *loci*.^(12)^ Besides, there are four major environmental exposures that could affect the rate of YCM in male offspring, such as the use of ICSI, the use of spermatozoa from PESA, vasectomy and its duration, and the age of the fathers. The data reported in this study observed only two of the four exposures, that is, the use of spermatozoa from PESA and vasectomy reversal. Maybe, future studies will consider the major environmental exposures in a larger sample.

A positive feature of the present study is the thorough evaluation of Y chromosome deletions assessing 20 regions of the chromosome, instead of the six regions usually considered in the clinical routine, and in most studies on the subject, thereby providing an increased sensitivity to detect this deletion (partial or complete YCM). Sachdeva et al.^(26)^ demonstrated that the incidence of YCM in infertile patients increases from 3% when evaluating 6 STS to 10.5% when evaluating 20 STS. Moreover, by screening for more Y-chromosome regions is possible to observe partial AZF deletions.

Another advantage of this study is the sperm analysis in the Control Group of fertile men, since in many publications evaluating the YCM incidence in the fertile population, the spermogram was not taken into account. Moreover, some of the microdeletions found in the fertile population may be associated with impaired spermatogenesis, which, however, were not enough to cause infertility.

The alterations found in sperm analysis of the fertile men group can be interpreted in different ways, and may reflect an impairment in spermatogenesis after conception, transitory sperm changes caused by diseases or medications, or cases where fertility was achieved even when the semen parameters were below the standards established by the WHO.^(19)^ Thus, fertility proven only by parenthood alone cannot be regarded as evidence of normal spermatogenesis, and it should be associated with sperm counting with normozoospermia. The studies that found high incidences of YCM were based on proven fertility for conceiving children and not by normal sperm analysis, which can cause false high incidences.^(14,27)^

The study of the YCM presence in the general population is gaining importance, because besides the proven association with abnormal spermatogenesis, these genetic abnormalities may be related to other consequences for the couple’s fertility, like in a situation with repeated abortions. Men whose partner had repeated miscarriages have higher incidence of microdeletions of the Y chromosome, quantified in 16%.^(28)^

The Control Group is one of the largest obtained in the literature so far, and the YCM incidence was similar to that found in Control Groups formed in several studies of these deletions in infertile population, ranging from 0 to 0.87%.^(14,23,24,29)^

This research showed that age at the time of vasectomy in groups that underwent vasectomy reversal, or IVF with PESA, was significantly lower than the mean of the Control Group. This fact is corroborated by the literature, reporting patients who underwent vasectomy at younger ages have a higher chance of searching for fertility recovery.^(3)^

One limitation of the study is the relatively low number of cases presented in groups of patients undergoing vasectomy reversal and IVF with PESA. Despite the large number of patients undergoing fertility recovery procedures that have been considered initially, obtaining patients for the study was greatly restricted. In addition, the low incidence of microdeletions in normal population restricts the conclusions and statistical analysis based on small and medium sized samples.

Yet, the study does not lose validity and, based on the observed incidence and on sample size, it is clear that the incidence of microdeletions in the presented groups is very low, being quite probably within the population mean. Furthermore, by the samples obtained, it is very unlikely that the studied populations present incidence of microdeletion as high as 10%, according to a previous study.^(12)^

New methods for detection of YCM are being proposed in an attempt to increase the efficiency and sensitivity of the method. Sun et al. demonstrated the efficacy of suspension array method in order to detect YCM, showing good correlation with the traditional PCR method used, and obtaining quicker results.^(23,24,30)^

It is possible that the investigation of YCM presence in other cells increase the sensitivity of the method. Some studies showed that the incidence of YCM is greater when measured in spermatic DNA and when compared to blood DNA. This higher incidence may be associated with teratozoospermy cases. The incidence observed in sperm of infertile men was 12.9%.^(29,31)^

## CONCLUSION

In summary, the incidence of Y-chromosome microdeletions in individuals born from vasectomized fathers who have undergone vasectomy reversal, or *in vitro* fertilization with spermatozoa retrieved by percutaneous epididymal sperm aspiration, did not differ between groups. This fact was not different from control subjects born from natural pregnancies, or from population incidence in fertile men, suggesting that the assisted reproduction techniques do not increase the incidence of Y chromosome microdeletion.
